# Temporal Ordering of Cancer Microarray Data through a Reinforcement Learning Based Approach

**DOI:** 10.1371/journal.pone.0060883

**Published:** 2013-04-02

**Authors:** Gabriela Czibula, Iuliana M. Bocicor, Istvan-Gergely Czibula

**Affiliations:** Department of Computer Science, Babes-Bolyai University, Cluj-Napoca, Romania; Queen's University Belfast, United Kingdom

## Abstract

Temporal modeling and analysis and more specifically, temporal ordering are very important problems within the fields of bioinformatics and computational biology, as the temporal analysis of the events characterizing a certain biological process could provide significant insights into its development and progression. Particularly, in the case of cancer, understanding the dynamics and the evolution of this disease could lead to better methods for prediction and treatment. In this paper we tackle, from a computational perspective, the *temporal ordering* problem, which refers to constructing a sorted collection of multi-dimensional biological data, collection that reflects an accurate temporal evolution of biological systems. We introduce a novel approach, based on reinforcement learning, more precisely, on *Q-learning*, for the biological temporal ordering problem. The experimental evaluation is performed using several DNA microarray data sets, two of which contain cancer gene expression data. The obtained solutions are correlated either to the given correct ordering (in the cases where this is provided for validation), or to the overall survival time of the patients (in the case of the cancer data sets), thus confirming a good performance of the proposed model and indicating the potential of our proposal.

## Introduction

The progresses from the last decades in the field of biology have resulted in an exponential increase in the amount of biological information. Depending on the type and purpose of biological experiments, the gathered data may vary from nucleotide or protein sequences, structures or functions, to molecular interactions and metabolic pathways. Analysis of this data reveals important insights into different biological processes and eventually leads to a better understanding of living organisms.

Biological processes are mostly dynamic and therefore, in order to accurately characterize them, scientists need dynamic information. However, most existing data is static, because it is often more difficult and challenging to follow a certain process over its full development. For instance, in the case of a disease, in certain situations it is only possible to extract data from a current pool of patients, rather than following the same patients over the full course of the disease. Therefore, the need to extract dynamic information from static data appears and a possible way of achieving this goal would be to infer temporal orderings to this data.

In this paper we tackle, from a computational perspective, the biological *temporal ordering (TO) problem*, which refers to constructing a sorted collection of multi-dimensional biological data, collection that reflects an accurate temporal evolution of a certain biological process. Cell division and growth, development, cell lineage, metabolism, or, more particular, certain classes of diseases (like cancer) are just some examples of such dynamical biological processes. The multi-dimensional input data may be the result of various biological experiments: protein expression, DNA microarrays, SNP arrays, chromosomal copy number alterations, comparative genome hybridization. In this work, we restrict to considering data sets consisting of samples derived from microarray gene expression experiments.

The *temporal ordering problem* addressed in this paper will be defined in the following, and the importance of the problem will be emphasized. We also present several related approaches for solving the TO problem, already existing in the literature.

### The Problem Statement and Relevance

Temporal modeling and analysis and more specifically, temporal ordering is an important research direction within multiple fields. From a machine learning perspective, in many situations, ordering a given data set of instances in time provides more significant information than assigning them to certain classes. Therefore, the general problem of temporal ordering is comparable, as importance, to the classification problem [1].

Within the bioinformatics and computational biology framework, the temporal ordering problem can be expressed in various forms. One definition of this problem refers to determining and describing the sequence of events that characterize a biological process. If the process in question is cancer, for instance, the goal is to find a temporal order for the genetic and pathway alterations that occur during the genesis and evolution of this disease. It is known that most tumors develop on account of malfunctioning of the complex signaling networks, which is the result of mutations that appear in certain key genes (oncogenes or tumor suppressor genes) [2]. Therefore, studying the order in which these mutations happen could lead to a better understanding of the evolution of cancer. Several works exist in the literature that approach the temporal ordering problem as it was described above and these will be presented in the following subsection.

The temporal ordering problem can also be formulated as the problem of constructing a sorted collection of multi-dimensional biological data, collection that reflects an accurate temporal evolution of a certain biological process. The final goal is to find certain patterns in the input data that vary over time and use them efficiently in order to be able to offer a proper characterization of the process in question. In what concerns this direction of study, there are mainly two works that have approached this problem and these will also be discussed in the following subsection. We mention that we tackle the temporal ordering problem, formulated in this second manner.

One of the most significant applications of this problem is within the field of cancer research. The majority of human cancer experiments provide data with no temporal information, because often it is too difficult, or even impossible, to follow the same patients over the full development of the disease. Instead, experimental samples are collected from current pools of patients, whose diseases are at different stages of advancement and consequently each sample reflects a different degree of cancer progression. The construction of a correct temporal series of these samples could, on the one hand, provide meaningful information about the complex process of cancer evolution. On the other hand, the temporal order could be used for the prediction of survival times of newly diagnosed patients: assuming that for the patients in the initial input data set survival times would be provided, when new patients, with unknown survival times are added to the data set, the recovered temporal order for the entire set of samples (including the newly added ones) could offer information on the overall life expectancies of the new patients.

### Literature Review

The general TO problem is known to be NP-complete [1], meaning that exact solutions are very difficult to obtain and therefore various heuristic methods have been applied to solve it. The general problem has mostly been approached by researchers of the artificial intelligence community (machine learning, data mining) [1], [3]. Within the data mining field, there are many studies that extract temporal information from different types of texts (general, medical, newspaper articles) [4]–[7]. Other applications include sorting photos of cities in order to observe their development over time [8] or building archaeological chronologies from various artefacts [9].

From the point of view of bioinformatics and computational biology, different forms of the TO problem have been studied and a significant number of researches focus on various forms of cancer. Due to the fact that this disease is an evolutionary process, which is driven by mutations and alterations of cell behaviour [10], one important line of work deals with developing models and inferring temporal orders to describe changes in cancer cells DNA as well as to determine the order in which gene mutation events and pathway variations happen during the evolution of cancer.

Several probabilistic models have been proposed in order to retrieve the temporal and casual order in which mutations happen on the level of genes and pathways, during cancer progression [10]–[12]. In the work of Hjelm *et al.* [11], the goal is to study chromosomal evolution in cancer cells by introducing and using graphical generative probabilistic models. Gerstung *et al.* [10] propose a probabilistic model based on bayesian networks, more specifically on a class of graphical models called Hidden Conjunctive Bayesian Networks (H-CBNs), which were previously proposed to study the accumulation of mutations and their interdependencies in cancer progression [12]. The tests were made on data sets containing cross-sectional mutation data belonging to different types of cancer (colorectal, pancreatic and primary glioblastoma) and the conclusions are that these H-CBNs provide an intuitive model of tumorigenesis [10].

A different approach to this problem is based on builduing tree models of possible gene mutation events [13]–[17]. Desper *et al.* [13], [14] propose a tree model for oncogenesis and by using comparative genome hybridization data they show that, under certain assumptions, their algorithm infers the correct tree of events (where an event is seen as a loss or a gain on a certain chromosome arm). Their approach is based on the idea of a maximum-weight branching in a graph. This proposed methodology was further developed by Beerenwinkel *et al.*, whose model include multiple oncogenetic trees, corresponding to multiple temporal sequences of events that can lead to cancer [15], [16]. Pathare *et al.* [17] analyze oral cancer progression using both models: distance trees introduced by Desper *et al.* [14] and the mixture of oncogenetic trees introduced by Beerenwinkel *et al.* [15], [16].

Mathematical approaches have also been proposed to tackle the problem of identifying the temporal sequence of mutations leading to cancer progression [18], [19]. Attolini *et al.* [18] introduce an evolutionary mathematical approach called Retracing the Evolutionary Steps in Cancer (RESIC), in order to identify the temporal order of gene mutations in cancer development and they test it on several colorectal cancer, glioblastoma and leukemia data sets. This method was further developed in [19] in order to incorporate, besides genetic alterations, modifications of the molecular signaling pathways by which cancer progresses.

Another important research direction focuses on a different formulation of the TO problem. Within this line of work, the problem is to construct a sorted collection of multi-dimensional biological data that reflects an accurate temporal evolution of a biological process. We tackle the TO problem from the point of view of this second definition. To our knowledge, there are mainly two works that approach the biologiocal TO problem as formulated above, both of them using gene expression data obtained from microarray experiments. These will be briefly presented in the following.

The first technique, which uses cancer gene expression data, is introduced by Gupta and Bar-Joseph [20]. The authors formally prove that, under certain biological assumptions on the input data set, the unique solution of the traveling salesman problem (TSP) represents the correct temporal ordering, with a high probability. The TSP is defined using the samples composing the input data set, which are characterized by multi-dimensional gene expression data, as vertices and the distances between them are computed using the Manhattan (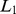) metric. The method is applied on a data set of 50 glioma patients and the results show a good correlation with the survival duration of the patients. Furthermore, a classifier that uses the obtained ordering is defined, which proves to outperform other classifiers developed for the considered task and key genes that are associated to cancer are identified.

The second study that approaches this form of the biological TO problem is introduced by Magwene *et al.* [21] and the proposed method is based on minimum spanning trees and PQ-trees. The minimum spanning tree algorithm is applied on a weighted, undirected graph, where each node is represented by one instance of the data set, represented by multi-dimensional microarray data. The efficacy of this method is proven by testing the algorithms on artificial data sets, as well as on time-series gene expression data sets derived from DNA microarray experiments.

The main contribution of this paper is that it introduces a novel approach to the TO problem, formulated as the problem of constructing a sorted collection of multi-dimensional biological samples, based on reinforcement learning. Reinforcement learning [22] is an approach to machine intelligence in which an agent [23] can learn to behave in a certain way by receiving punishments or rewards on its chosen actions. To the best of our knowledge, the TO problem has not been addressed in the literature using reinforcement learning, so far. Several experiments performed on different DNA microarray data sets show that the proposed reinforcement learning based approach successfully identifies accurate temporal orderings of the given biological samples.

## Methods

In this section we introduce our reinforcement learning based proposal for identifying a temporal ordering of a series of biological samples. Even though in this study we restrict to gene expression data obtained from microarray experiments, the applicability of our method is more general and it can be used with different types of multi-dimensional biological data.

We start by presenting the fundamentals of *reinforcement learning*, then we detail our approach.

### Reinforcement learning. Background

The goal of building systems that can adapt to their environments and learn from their experiences has attracted researchers from many fields including computer science, mathematics, cognitive sciences [22]. *Reinforcement Learning* (RL) [24] is an approach to machine intelligence that combines two disciplines to successfully solve problems that neither discipline can address individually: *Dynamic programming* and *Supervised learning*. In the machine learning literature, RL is considered to be the most reliable type of learning, as it is the most similar to human learning.

Reinforcement learning deals with the problem of how an autonomous agent that perceives and acts in its environment can learn to choose optimal actions to achieve its goals [25]. The field of *intelligent agents* [26] is an important research and development area in the artificial intelligence field, agents being considered new important means in conceptualization and implementation of complex software systems. An agent is a computational entity such as a software system or a robot, situated in a certain environnment, that is able to perceive and act upon its environment and is capable to act autonoumously in order to meet its design objectives. Agents are acting in behalf of users, are *flexible* [27], meaning that they are *reactive* (able to respond to changes that occur in their environment), *pro-active* (able to exhibit goal directed behavior) and also have a *social ability* (are capable of interacting with other agents).

Reinforcement learning is useful in a lot of practical problems, such as learning to control autonoumous robots [28], learning to optimize operatons in factories or learning to play board games. In all these problems, an artificial agent has to learn (by reinforcement) to choose optimal actions in order to achieve its goals.

In a reinforcement learning scenario, the learning system selects actions to perform in the environment and receives *rewards* (or *reinforcements*) in the form of numerical values that represent an evaluation of the selected actions [29]. In RL, the computer is simply given a goal to achieve. The computer then learns how to achieve that goal by trial-and-error interactions with its environment. Reinforcement learning is learning what to do - how to map situations to actions - so as to maximize a numerical reward. The learner is not told which actions to take, as in most forms of machine learning, but instead must discover which actions yield the highest reward by trying them. In a reinforcement learning problem, the agent receives the reward as a feedback from the environment; the reward is received at the end, in a terminal state, or in any other state, where the agent has correct information about what he did well or wrong. The agent will learn to select actions that maximize the received reward.

The agent's goal, in a RL task is to maximize the sum of the reinforcements received when starting from some initial state and proceeding to a terminal state.

A reinforcement learning problem has three fundamental parts [22].

*The environment* is represented by “states”. By interactions with the environment, a RL system will learn a function that maps states to actions.

*The reinforcement function*. The goal of the reinforcement learning system is defined using the concept of a reinforcement function, which is the function of reinforcements the agent tries to maximize. This function maps state-action pairs to reinforcements. After an action is performed in a certain state, the agent will receive an evaluation of the action in a form of a scalar reward. The agent will learn to perform those actions that will maximize the total amount of reward received on a path from the initial state to a final state [30].

*The value (utility) function* is a mapping from states to state values. The value of a state indicates the desirability of the state and is defined as the sum of rewards received on a path from that state to a final state. The agent will learn to choose the actions that lead to states having a maximum utility [30].

A general RL task is characterized by four components:

1. a *state space* 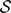 that specifies all possible configurations of the system;
2. an *action space* 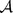 that lists all available actions for the learning agent to perform;
3. a *transition function* 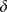 that specifies the possibly stochastic outcomes of taking each action in any state;
4. a *reward function* that defines the possible reward of taking each of the actions.

At each time step, 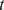, the learning system receives some representation of the environment's state 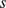, it takes an action 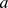 and one step later it receives a scalar reward 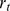 and finds itself in a new state 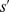. The two basic concepts behind reinforcement learning are trial and error, search and delayed reward [31]. The agent's task is to learn a control policy, 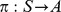, that maximizes the expected sum 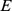 of the received rewards, with future rewards discounted exponentially by their delay, where 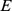 is defined as 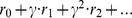 (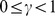 is the discount factor for the future rewards).

An important aspect in reinforcement learning is the *exploration*. The agent has to be able to explore its environment, by trying new actions (maybe not the optimal ones) that may lead to better future action selections [32].

There are two basic RL designs to consider:

- The agent learns a *utility function* (*U*) on states (or states histories) and uses it to select actions that maximize the expected utility of their outcomes.
- The agent learns an *action-value function* (*Q*) giving the expected utility of taking a given action in a given state. This is called *Q-learning*.

An agent that learns utility functions [33] must have a model of the environment in order to make decisions, as it has to know the states to which its action will lead. In a *Q-learning* scenario, in which the agent learns an action-value function, there is no need to have a model of the environment.

### Our approach. Methodology

Let us consider, in the following, that 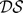 is the input data set, consisting of 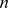 (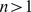) multi-dimensional samples: 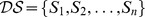, each sample being identified by a set of features. For the considered type of data, each feature is represented by one gene and has as a value a real number, measuring the expression level of the gene in question. Therefore, every sample may be encoded by an 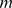-dimensional vector 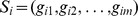, where 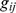 is the expression level of gene 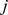 for the sample 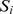.

Our approach consists of two steps:

1. Data pre-processing.
2. *RL* task design.

In the following we will describe these steps.

#### Data pre-processing

DNA microarrays allow measuring of thousands of gene expression levels for each sample, thus the dimensionality of the input data can be extremely high. Besides the fact that this might lead to inefficiency in computational time and space, in most cases, many genes can be irrelevant for the ordering task and can even increase the amount of noise in the data, leading to a decrease in the performance of the temporal ordering system. Therefore, the goal of the pre-processing step is the elimination of the genes that offer no significant information, or, equivalently, the selection of those genes that are most important for an accurate temporal ordering.

As the final goal consists in analyzing and temporally ordering data sets comprising samples extracted from cancer patients, in the following, we describe a pre-processing method targeting these particular types of data sets. Such data sets usually offer a series of information for each sample, besides the actual gene expression vectors. One of these extra pieces of information that is regularly found in cancer data sets is the overall survival, meaning the survival time of the patients, following the moment in which the samples were taken. Starting from the intuition that, in the general case, two patients having similar survival times would also be relatively close within the temporal ordering, we decided to use this piece of information for identifying a subset of genes that are relevant for the ordering task.

During the pre-processing step, a statistical analysis is carried out on the data set 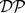 in order to find a subset of features (genes) that are relevant for the considered task. The statistical analysis on the features is performed in order to reduce the dimensionality of the input data, by eliminating features that are not correlated with the selected extra biological information for the given data set. More exactly we aim at identifying genes that do not significantly influence the temporal ordering identification.

To determine the dependencies between the features and the given additional biological information, the Pearson correlation coefficient is used [34]. The Pearson correlation is a statistical measure of the linear correlation between two random variables indicating how highly correlated the variables are. A Pearson correlation of 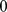 between two variables 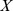 and 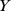 indicates that there is no linear relationship between the variables. A Pearson correlation of 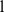 or 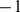 results when the two variables being compared are linearly monotonically related. A Pearson correlation [35] of 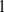 implies that a linear equation describes the relationship between 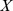 and 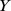, with all data points lying on a line for which 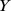 increases as 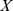 increases. A correlation of 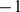 implies that all data points lie on a line for which 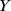 decreases as 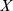 increases.

As mentioned before, the goal of this step is to remove from the feature set those features (genes) which are very slightly correlated with the selected supplementary biological information (which is, in the case of cancer data sets, the survival time). Consequently, we compute the Pearson correlation coefficient between each gene and the survival time and we keep only those genes which have the absolute value of the correlation greater than a certain threshold 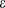 (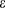 is chosen so as to ensure a radical reduction of dimensionality).

#### The proposed *RL* task for the TO problem

As indicated above, the TO problem consists of determining an accurate temporal ordering of the input samples, which would reflect the temporal evolution and development of a certain dynamical biological process (e.g. cancer). From a computational point of view, the TO problem can be viewed as the problem of generating a permutation 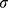 of  that maximizes the overall similarity Sim of the sequence of samples considered in the order 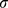: 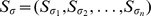 (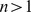). The overall similarity Sim we consider in this paper sums the similarities over all adjacent samples and it has to be maximized.

The overall similarity Sim for the sequence of samples 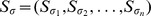 is defined as in Equation (1):
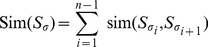
(1)where 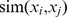 denotes the similarity between the 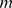-dimensional vectors 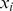 and 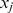 and is defined as 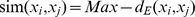. Here by 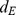 we denote the euclidian distance and 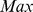 is a large constant.

We define the RL task associated to the TO problem as follows:

- The state space 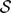 (the agent's environment) will consist of 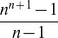 states, i.e. 
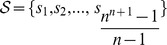
. The *initial state* of the agent in the environment is 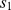. A state 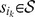 
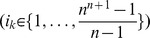
 reached by the agent at a given moment after it has visited states 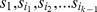 and has selected actions 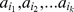 is a *terminal* (final or goal) state if the number of states visited by the agent in the current sequence is 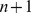 (i.e. 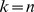) and all the selected actions are distinct, i.e. 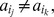 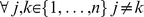.
- The action space 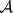 consists of 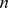 actions available to the problem solving agent and corresponding to the 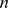 possible values 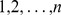 used to represent a solution (permutation of 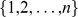), i.e. 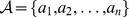, where 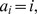 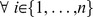.
- The transition function 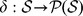 between the states is defined as in Formula (2).
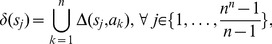
(2)where 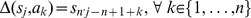. This means that, at a given moment, from a state 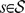 the agent can move in 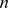 successor states, by executing one of the 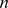 possible actions. We say that a state 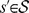 that is accessible from state 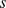, i.e. 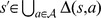, is the *neighbor* (*successor*) state of 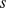.

The transitions between the states are equiprobable, the transition probability 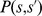 between a state *s* and each neighbor state 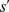 of 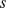 is equal to 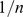, as each state from 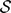 has 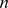 possible successor states (see Formula (2)).

- The reward function will be defined below (Formula (3)).

Let us consider a path 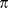 in the above defined environment from the initial to a final state, 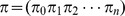, where 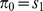 and 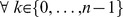 the state 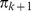 is a *neighbor* of state 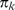 (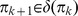). Considering the RL task defined above, the environment may be visualized as a tree. In this tree-like environment, a path 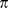 consists of distinct vertices (states) in which each adjacent pair of vertices is linked by an arc (action).

The sequence of actions obtained following the transitions between the successive states from the path 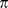 will be denoted by 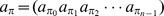, where 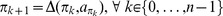. The sequence 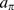 will be referred to as the *action configuration* associated to the path 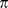. The *action configuration* associated to a path 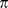 gives a sequence of samples 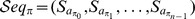.

A path 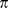 is called *valid* if all the actions within its *action configuration* are distinct and each sample 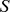 from the sequence 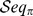 is more similar to the sample that immediately follows it in the ordered sequence than to any other sample, i.e. 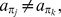 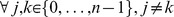 and 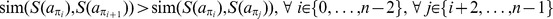.

The *action configuration* 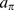 associated to a *valid* path 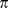 can be viewed as a possible order for the input samples, i.e. a permutation that gives the temporal ordering 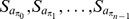 of the considered samples, which should be, to a certain degree, correlated with the survival time, in the case when the samples are represented by data extracted from cancer patients. Consequently, we can associate to a *valid* path 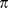, a value denoted by 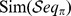 representing the overall similarity (see Equation (1)) of the sequence 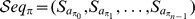.

The TO problem formulated as a RL problem will consist of training the agent to find a path 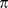 from the initial to a final state having the maximum associated overall similarity 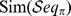. After the reinforcement learning process, the agent will learn to execute those transitions that maximize the sum of rewards received on a path from the initial to a final state.

We aim at obtaining a *valid* path 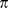 having the maximum overall similarity of the sequence of samples corresponding to the associated action configuration 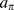, hence we define the reinforcement function as follows (Formula (3)):
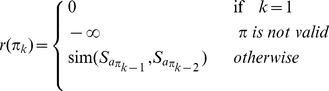
(3)where by 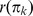 we denote the reward received by the agent in state 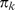, after its history in the environment is 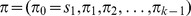.

The agent receives a negative reward on paths that are not valid, therefore it will learn to explore only valid paths. Considering the reward defined in Formula (3), as the learning goal is to maximize the total amount of rewards received on a path from the initial to a final state, it can be shown that the agent is trained to find a valid path 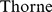 that maximizes the overall similarity of the associated ordering.

#### The learning process

During the training step of the learning process, the agent will determine its *optimal policy* in the environment, i.e. the the mapping from states to actions that maximizes the sum of the received rewards.

For training the *TO agent*, we propose a 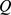-learning approach, in which the agent learns an action value function, denoted by 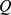, function giving the expected utility of taking a given action in a given state [22]. The idea of the training process is the following. After the 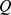 values are initialized, during some training episodes, the agent will experiment (using an action selection mechanism) some (possible optimal) *valid* paths from the initial to a final state, updating the 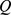-values estimations according to the 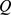-learning algorithm [36]. At the end of the training process, the 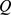-values estimations will be in the vicinity of the exact values. 
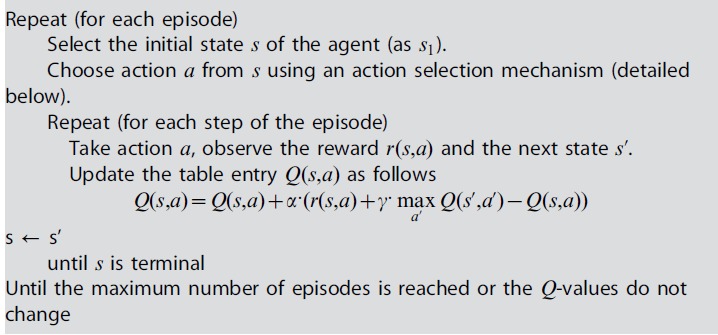


The general form of the 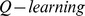 algorithm is given in Algorithm 1. We denote in the following by 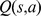 the Q-value estimate associated to the state 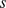 and action 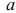, by 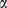 the learning rate and by 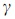 the discount factor.

### Algorithm 1. The Q-learning algorithm

The action selection mechanism we have used in the proposed 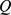-learning algorithm is derived from the 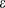-Greedy mechanism [22] and it uses a one step look-ahead procedure in order to guide the exploration of the search space. When selecting an action from a given state 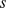, the following selection mechanism is used:

#### 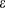-Greedy selection

With probability 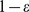 select the action 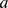 that maximizes the 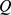-value of the reached neighboring state, i.e. 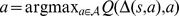.

#### Look-ahead

With probability 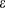, select the action 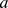 that gives the maximum total similarity of the action configuration corresponding to the current path.

After the training step of the agent has been completed, the solution learned by the agent is constructed by starting from the initial state and following the *Greedy* mechanism until a solution is reached. From a given state 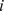, using the *Greedy* policy, the agent transitions to a neighbor 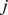 of 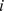 having the maximum 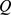-value. Consequently, the solution of the TO problem reported by the RL agent is a path 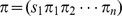 from the initial to a final state, obtained following the policy described above. It has been proven that the learned 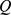-values converge to their optimal values as long as all state-action pairs are visited an infinite number of times [37]. Consequently, the action configuration 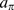 corresponding to the path 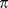 learned by the TO agent converges, in the limit, to the optimal time ordering of the samples, 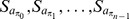, having the maximum associated overall similarity.

## Results

In this section we aim at experimentally evaluating our *RL*-based approach for solving the TO problem. For the computational experiments developed in order to test the performance of our method, we firstly used an artificially generated data set and then we continued our experiments on several real data sets, which were chosen for the following reasons:

- They are publicly available.
- They resulted from different types of biological experiments (yeast cells affected by environmental changes [38], *Saccharomyces cerevisiae* yeast 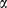-factor based synchronization [39], human cells responding to infection by a bacterial pathogen [40], brain tumor gene expression data [41]).
- They are either time series (therefore the correct order is known) or they contain survival time information (corresponding to each sample), which gives us the possibility to validate our results.
- They have been used in other works which tackled the time ordering problem, thus allowing us to compare our results with other results existing in the literature.

### Synthetic Data

First, in order to test the ability of our algorithm to determine accurate orderings, we used synthetic data obtained in the following way. We have artificially generated a sample data set consisting of 10 samples, 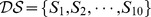. Table 1 indicates the randomly generated survival times (in days) associated to the samples and Table 2 illustrates the similarities between the samples. The similarity measures were generated so as to be consistent with the given survival times.

**Table 1:** The artificially generated survival times associated to the samples.

		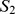	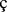		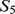	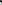	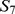	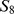	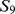	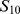
**Survival time (days)**	400	650	60	532	125	21	200	480	310	100

Randomly generated survival times (in days) associated to the samples from the synthetic data set.

**Table 2:** The similarity scores for the samples from the synthetic data set.

similarity	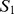	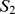	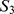	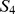	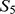	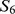	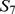	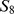	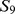	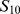
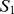	–	4	3.67	3.34	2.68	3.67	5	5	6	3.67
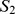	4	–	2.67	5	2.34	2.67	3.34	7	4.01	3.01
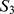	3.67	2.67	–	1.67	5	7	4.34	3.67	3.34	7
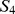	3.34	5	1.67	–	1.67	1.68	3.68	5.01	3.34	2.35
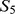	2.68	2.34	5	1.67	–	4	5	2.34	4	6
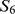	3.67	2.67	7	1.68	4	–	3.34	3.67	3	6
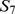	5	3.34	4.34	3.68	5	3.34	–	3.68	7	3
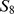	5	5	3.67	5.01	2.34	3.67	3.68	–	3.34	5.01
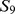	6	4.01	3.34	3.34	4	3	7	3.34	–	3.34
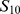	3.67	3.01	7	2.35	6	6	3	5.01	3.34	–

Using the values indicated in Table 2, considering the scores between the samples as a measure of their similarity, we apply our previously introduced RL approach in order to find a *valid* ordering of the samples 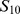 having a maximum associated *overall similarity* (see Equation (1)).

The maximum value for the overall similarity Sim of *valid* solutions is 53.01 and it is obtained in two cases: for the orders 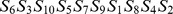 and 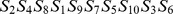. The first order is the one that is correlated with the generated survival times. The solutions were obtained using a backtracking algorithm and are used for evaluating the RL result.

#### RL model and results

For the example presented in this section, we built the states and action spaces of our RL model, in order to be able to identify the temporal ordering that is correlated with the survival time, i.e. the valid ordering that maximizes the overall similarity. The states space will consist of 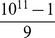 states, while the action space will contain 10 actions, corresponding to the 10 given samples.

For applying the RL model introduced above, we have used a software framework that we have previously introduced for solving combinatorial optimization problems using reinforcement learning techniques [42].

We have trained the TO agent as indicated in the Methods section of this paper. We remark the following regarding the parameters setting: the discount factor for the future rewards is 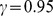; the learning rate is 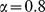; the number of training episodes is 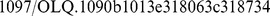; the modified 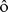-Greedy action selection mechanism was used with 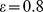. The solution reported after the training of the TO agent was completed is the optimal valid ordering, it has the maximum associated overall similarity of 53.01 and was determined starting from state 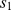, following the *Greedy* policy. The learned solution is the temporal ordering that is correlated with the survival time, that is the path having the associated *action configuration* 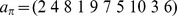. Here, we remark that the algorithm may recover the correct ordering, or its reverse, as it has no way of determining which end of the obtained permutation is actually the first sample. The correct first sample is subsequently chosen using the given survival time. The solution was obtained in less than 2 seconds on a PC at 3 GHz with 4 GB of RAM.

**Figure 1:**
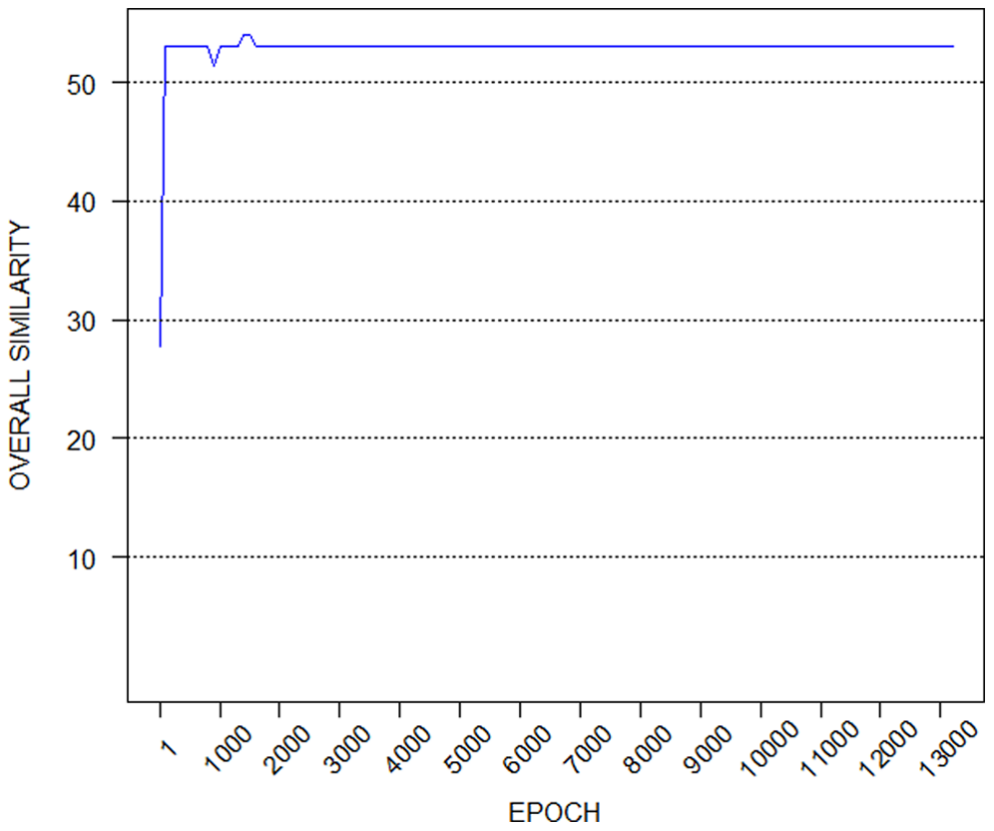
Synthetic data set results: the learning process. Illustration of the overall similarity of the solutions obtained during the training process, from 100 to 13000 training epochs. It can be seen how, during the training, the learned solution converges to the optimal one.

Figure 1 depicts the overall similarity of the solutions obtained during the training process, from 100 to 13000 training epochs. It can be seen how, during the training, the learned solution converges to the optimal one.

**Figure 2:**
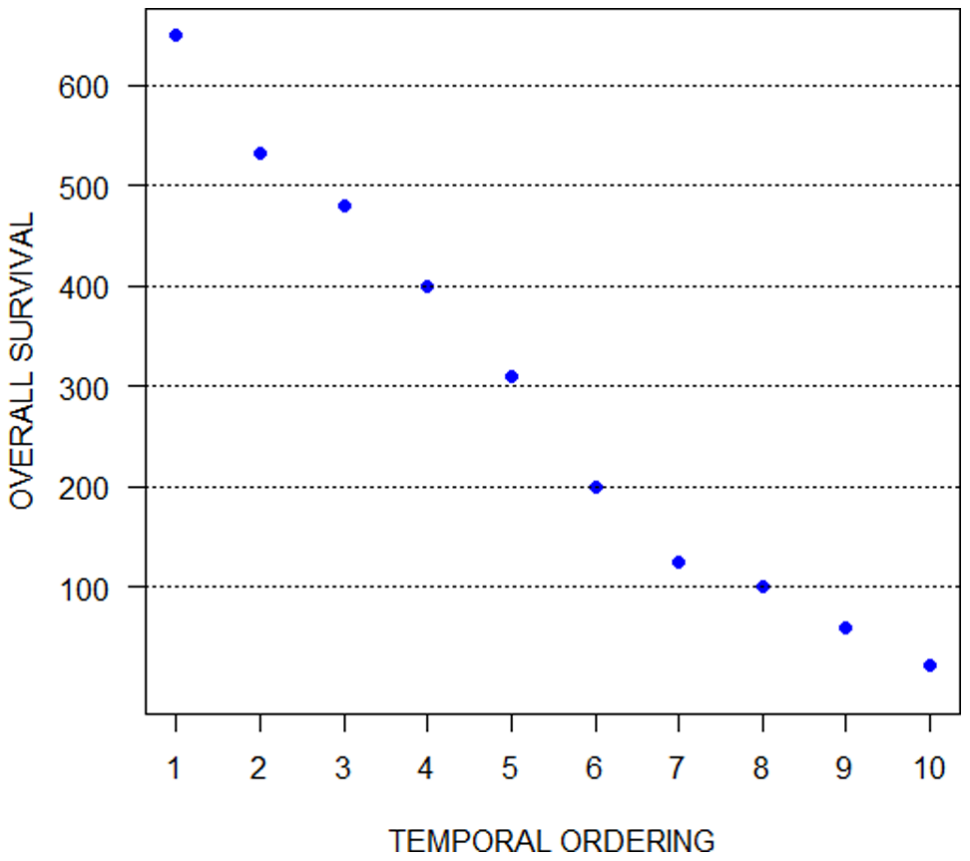
Synthetic data set results: the recovered ordering and the corresponding survival times. The ordering recovered by our algorithm agreed well with the survival time following the point when samples were taken.

The ordering recovered by our algorithm agreed well with the survival time following the point when samples were taken. Figure 2 presents the duration patients survived for the identified ordering.

### Time Series Gene Expression Data

In order to test our method on data with known time orderings, we used several time series data sets. A time series data set is a collection of data resulted from a specific type of biological experiment: samples of tissues are extracted from the same individual at different, known points in time, during the progression of the biological process. Thus, for a time series data set, the exact time of each sample is provided and therefore the ordering is known.

We present, in the following, several time series data sets and the results we obtained after applying the proposed RL algorithm. We mention that, for all the data sets we excluded those genes that had missing expression levels for certain time points and then we applied the pre-processing procedure that we previously described, with a slight modification. Instead of computing the Pearson correlation coefficient between each feature (gene) and the survival time (which is inexistent information for the time series case), we use the available information (the given exact points in time of each sample) in order to compute the correlation. As threshold value, we considered 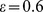 and therefore all the genes that had the absolute value of the Pearson correlation below 0.6 were removed. The threshold value was chosen rather high because we intended to significantly reduce the dimensionality of the data.

#### Yeast Time Series Gene Expression Data

We conducted the first series of experiments on five data sets, composed of gene expression data measuring the levels of expression of almost every yeast gene, as yeast cells were affected by various environmental changes [38]. The authors tested the response of yeast cells for several environmental stresses [38], from which we only consider five conditions: heat shock, DTT exposure, amino acid starvation, nitrogen depletion and diauxic shift. For each of these, sampling was made at a different number of points in time and at different periods of time, this being illustrated in Table 3.

**Table 3:** Time series data sets.

**Yeast cells affected by environmental changes ** [**38**]		
**Environmental condition**	**Number of time points**	**Sampling period**
Heat shock	8	5, 10, 15, 20, 30, 40, 60, 80 (min)
DTT exposure	8	5, 15, 30, 45, 60, 90, 120, 180 (min)
Amino acid starvation	5	0.5, 1, 2, 4, 6 (h)
Nitrogen depletion	10	0.5, 1, 2, 4, 8, 12, 24, 48, 72, 120 (h)
Diauxic shift	7	9.5, 11.5, 13.5, 15.5, 18.5, 20.5 (h)
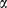 **factor-based synchronization of the ** ***Saccharomyces cerevisiae*** ** yeast cells ** [**39**]		
	**Number of time points**	**Sampling period**
	18	7, 14, 21, 28, 35, 42, 49, 56, 63, 70, 77, 84, 91, 98, 105, 112, 119, 126 (min)
**Response of human cells to infection by ** ***Listeria monocytogenes*** **** [**40**]		
**Condition**	**Number of time points**	**Sampling period**
Wild type 1	6	0, 30, 60, 120, 240, 480 (min)
Wild type 2	6	0, 30, 60, 120, 240, 480 (min)
Mutant 1	6	0, 30, 60, 120, 240, 480 (min)
Mutant 2	6	0, 30, 60, 120, 240, 480 (min)

Illustration of the yeast and human time series data sets: the condition the cells were exposed to, the number of time points and the sampling period, in minutes (min) or hours (h).

Next, we tested our algorithm on a different yeast time series data set, described by Spellman *et al.* [39]. The authors present several experiments conducted with the aim of identifying cell-cycle regulated genes of the yeast *Saccharomyces cerevisiae* [39]. Among these, a time series data set referring to 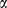 factor-based synchronization of the yeast cells is also described. The sampling was made at every seven minutes and samples were taken at eighteen time points, as illustrated in Table 3.

#### Human Time Series Gene Expression Data

Following the yeast time series, we continued to test the RL based algorithm on four human gene expression data sets, obtained by Baldwin *et al.* during the examination of the response of cultured human intestinal epithelial cells to infection by a bacterial pathogen (*Listeria monocytogenes*) [40]. Each data set is composed of gene expression data collected at six different time points and the experiments were made for the wild type, as well as for a bacterial mutant. These data sets are also presented in Table 3.

#### Evaluation measure

To be able to compare our results with other results presented in the literature for the same data sets, as well as in order to quantify the performance of our RL based algorithm, we introduce an evaluation measure which assesses the quality of a solution (ordering) obtained for a data set, with regard to the correct, known ordering. We define a measure, SMD (*Samples Misplacement Degree*), which, in our view, expresses the misplacement degree of samples in a given ordering (solution). Through its definition, the SMD measure penalizes solutions in which samples are not in the correct position in the ordering, with respect to neighboring samples. Hence, for a reported solution, we compute the associated evaluation measure as the number of *misplaced samples*. A sample is defined as being misplaced if its time point in the correct ordering is not included in the interval having as bounds the time points of the sample's neighbors from the obtained ordering.

Let us consider in the following that 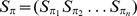 is an ordering of the 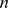 initial samples 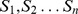, where 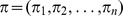 is a permutation of the set 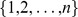. For each sample 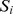, the time point of the sample is known and is denoted by 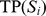.

#### Definition 1

(Samples Misplacement Degree - SMD.) The misplacement degree of samples in the sequence (ordering) 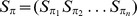, denoted by 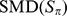, is defined as
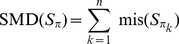
(4)
*where* 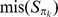 *is* 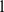 *if the sample* 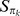 *is misplaced in the ordering and* 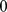 *otherwise*.

The misplaced function mis is defined as follows:

- As special cases, for the first and last samples in the ordering, we consider that the sample is misplaced if its time point is neither 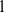 nor 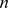, i.e. if 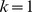 or 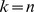 then
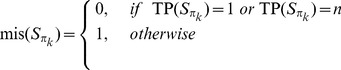
(5)- For the rest of the samples (excluding the first and the last ones, i.e. 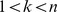) the misplaced function is defined as
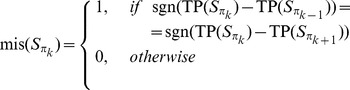
(6)By sgn we have denoted the *signum function*.

Based on Definition 1, it can be simply shown that 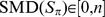. The evaluation measure SMD for the correct solution (known ordering) is zero, as in the correct ordering all the samples are correctly placed (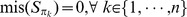). If there are misplaced samples in the sequence, then 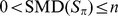.

Between two different orderings for the same data set, the best one will always be the one having a lower value of the evaluation measure. Consequently, smaller values for the SMD (smaller numbers of misplaced samples) indicate better orderings, this meaning that the SMD has to be minimized.

It can also be observed that the functions mis and SMD were defined so as to take into consideration the case in which the obtained ordering is the reverse of the correct one. For such a situation, the evaluation measure will also be zero, because the solutions are time-reversible (the algorithm cannot distinguish which of the two ends of the ordering is actually the first, without additional biological information).

### Experimental Results

For applying our RL based approach on the data sets presented above, we have used a software framework that we have previously introduced for solving combinatorial optimization problems using reinforcement learning techniques [42].

Concerning the RL parameters, we used the same values as for the synthetic data test: the discount factor for the future rewards is 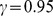; the learning rate is 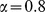; the modified 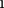-Greedy action selection mechanism was used with 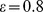; the number of training episodes is 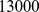. The solutions reported in each case, after the training of the TO agent was completed are the optimal valid temporal orderings and they were determined starting from the first state, following the *Greedy* policy.

For each of the time series data sets described above, Table 4 presents the solution obtained by our RL based temporal ordering algorithm, the evaluation measure SMD of the ordering, the computational time, as well as other orderings obtained in the literature for the same data sets and their corresponding evaluation measures. The last column of these tables specifies whether our method leads to better solutions (in terms of correct known ordering, or of lower values of the evaluation measure), compared to those that have already been reported in the literature.

**Table 4:** Results for the time series data sets.

**Yeast cells affected by environmental changes**						
**Data set**	**RL recovered ordering (S)**	**SMD (S)**	**Comp. time (sec.)** a	**Ordering recovered in literature (S')**	**SMD (S')**	**Imprv** b
Heat shock	1, 2, 3, 4, 5, 6, 7, 8	0	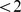	1, 8, 7, 6, 5, 4, 3, 2 [20]	2	Yes
DTT exposure	1, 2, 3, 4, 5, 6, 7, 8	0	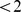	1, 2, 3, 4, 5, 6, 7, 8 [20]	0	Same
Amino acid starvation	1, 2, 3, 4, 5	0	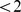	1, 2, 3, 4, 5 [20]	0	Same
Nitrogen depletion	4, 3, 2, 1, 5, 6, 7, 8, 9, 10	2	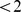	4, 3, 2, 1, 5, 6, 7, 8, 9, 10 [20]	2	Same
Diauxic shift	1, 2, 3, 4, 5, 6, 7	0	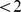	1, 2, 3, 4, 5, 6, 7 [20]	0	Same
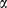 **factor-based synchronization of the ** ***Saccharomyces cerevisiae*** ** yeast cells**						
	1, 2, 3, 4, 5, 6, 7, 8, 9, 17, 14, 15, 16, 18, 10, 11, 12, 13	5	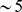	1, 2, 3, 4, 5, 6, 7, 8, 9, 10, 18, 17, 16, 15, 14, 13, 12, 11 [21]	2	No
**Response of human cells to infection by ** ***Listeria monocytogenes***						
Wild type 1	1, 2, 3, 4, 5, 6	0	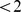	1, 2, 3, 4, 5, 6 [20]	0	Same
Wild type 2	1, 2, 3, 4, 5, 6	0	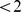	3, 2, 1, 5, 4, 6[20]	4	Yes
Mutant 1	1, 4, 2, 3, 5, 6	2	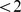	1, 3, 2, 6, 5, 4 [20]	4	Yes
Mutant 2	1, 2, 3, 4, 5, 6	0	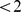	1, 2, 3, 4, 6, 5 [20]	2	Yes

Presentation of the results obtained by our RL based temporal ordering algorithm, the value of the evaluation measure SMD of the ordering, the computational time, other orderings obtained in the literature for the same data sets and their corresponding evaluation measures. The last column specifies specifies whether our method leads to better solutions (in terms of correct known ordering, or of lower values of the evaluation measure), compared to those that have already been reported in the literature.

a Computational time of our algorithm, in seconds.

b “Imprv.” is the abbreviation for “Improvement”, specifying whether our method obtained an improvement compared to the other methods existing in the literature.

We mention that for each of the ten data sets, the correct orderings are the ones starting with the first sample (corresponding to the sample extracted at the first point in time) and increasing consecutively up to the sample which was acquired last. It can be observed that our algorithm obtained the correct orderings for *seven* out of the ten data sets.

Regarding the yeast time series data taken from [38], we remark that our algorithm retrieved the correct time orderings for four out of the five considered experiments. For the case when the ordering was not correct (“nitrogen depletion”), the algorithm recovered two blocks, the internal ordering within each of these being correct. This behaviour could probably be explained by the fact that the first four samples have been harvested at very close periods in time (see Table 3), meaning that only very small changes were displayed with regard to the initial time point. This data set was also used in the study of Gupta and Bar-Joseph [20]. Out of the five yeast stress response time series, the results reported in [20] are correct for three cases. The TSP approach obtained the same (incorrect) ordering as our RL algorithm for the “nitrogen depletion” experiment, but for the “heat shock” experiment the results reported in [20] did not indicate the accurate ordering (but still retrieved two correctly internally ordered blocks), while our approach led to the correct result. Therefore, we may conclude that for this data set our RL based technique outperforms the TSP heuristic.

Our algorithm was less performant for the *Saccharomyces cerevisiae* yeast data set [39]. As can be seen in Table 4, the algorithm successfully separated the first and the second half of the ordering. Within the first block, the internal order is correct, but for the second half it is only partially accurate (samples 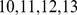). The results obtained by Magwene *et al.* [21] on the same data set are better: they do not retrieve the correct ordering, but their PQ-tree method separates the first and the second half of the time course, each half containing correctly internally ordered samples. These are also reflected in the values of the evaluation measure, as the ordering that we retrieved has a higher degree of misplacement of the samples (5) than the one retrieved in [21] (2).

For the human time series gene expression data set [40], the RL based approach that we proposed proved to obtain better orderings than the TSP heuristic presented in [20]. As can be seen in Table 4, our algorithm obtained the correct orderings in three out of the four cases (“Wild type 1”, “Wild type 2”, “Mutant 2”), while the TSP based algorithm retrieved the known accurate temporal orderings for only one case (“Wild type 1”). For the other three experiments, the TSP algorithm successfully separated the two halves of the time course, but the internal orderings are not all accurate. Concerning the experiment “Mutant 1”, neither our approach, nor the TSP method retrieved the correct ordering, but in terms of the evaluation measure that we have previously defined, our RL algorithm reported a slightly better solution.

Regarding the computational time, for the smaller data sets (those containing less than, or exactly 10 samples), our RL based algorithm obtained the solutions within very short amounts of time, less than 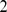 seconds, on a PC at 3 GHz with 4 GB of RAM, in all nine cases (Table 4). The computational time of our algorithm for the data set composed of 18 samples [39] was low as well - approximately 5 seconds (Table 4).

As shown in Table 4, the results that we obtained are comparable with the existing ones and in *four* cases they are better than the results that were reported, so far. Moreover, another advantage of our RL based approach is the low computational time.

### Cancer Expression Data

We also tested our RL based algorithm on a cancer gene expression data set [41], consisting of high-grade glioma samples: 28 glioblastomas and 22 anaplastic oligodendrogliomas. For each sample of these two subsets, we are given a series of information: the gene expression levels for 12625 genes, the vital status of each patient (alive or dead) and the survival time following the initial diagnosis or, for living patients, the survival is given to time of last follow-up [41].

For each of the two subsets (glioblastomas and anaplastic oligodendrogliomas), we tried to find a separate ordering. Before testing the RL algorithm, we applied the pre-processing step, meaning that the Pearson correlation coefficient was computed between each gene and the survival time, using the same threshold value as in the case of the time-series data sets: 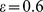. Following this step, the dimensionality of the input data was significantly reduced: the input vectors for glioblastoma reached a dimensionality of 28 features (genes), while those corresponding to anaplastic oligodendrogliomas were limited to 41 features. Feature dimensionality reduction methods have also been used in the original study providing the high-grade glioma samples [41], where the authors identify a total number of 20 features (10 for each type of glioma) which are highly correlated with the class distinction of either glioblastoma or anaplastic oligodendroglioma.

**Figure 3:**
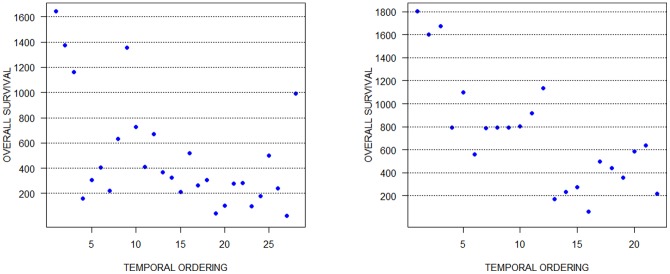
Recovered temporal orderings and survival times for the high-grade glioma data set. The figure on the left corresponds to the glioblastomas data set, while the one on the right illustrates the results for the anaplastic oligodendroglioma data set. It can be observed that, in both cases, the samples in the right half belong to patients whose survival times are lower, while the ones in the left half belong to patients having higher survival times.

The TO agent was trained using the following parameter setting: the discount factor for the future rewards is 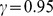; the learning rate is 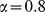; the number of training episodes is 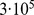; the modified 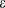-Greedy action selection mechanism was used with 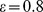. Following the training step, the ordering for each glioma subset was retrieved starting from the first state and following the *Greedy* policy. Figure 3 presents the obtained solutions and indicates the correlation between the orderings and the survival time of the patients. As mentioned before, the solutions are time-reversible: the algorithm cannot distinguish which of the two ends of the ordering is actually the first, therefore this can be determined using the additional biological information (the survival time). The orderings are illustrated so that the first sample is always on the left and the last one on the right. It can be observed that in both cases the recovered orderings are, up to a certain extent, well correlated with the overall survival: the samples in the right half of each graph belong to patients whose survival times are lower, while the ones in the left half belong to patients having higher survival times. This is also illustrated in Table 5, which shows that for both the glioblastomas and the anaplastic oligodendroglioma data sets the average survival time value of the left half is significantly higher than the average value of the right half. The last column of this table indicates the computational times of out RL algorithm. We mention that the experiments were conducted on the same hardware configuration, a PC at 3 GHz with 4 GB of RAM.

**Table 5:** Results for the high-grade glioma data sets.

Data set	Number of samples (time points)	Average of the left half of the ordering	Average of the right half of the ordering	Computational time (min)
Glioblastomas	28	696.64	288.14	∼30
Anaplastic oligodendrogliomas	22	1057.00	418.90	∼20

For both the glioblastomas and the anaplastic oligodendroglioma data sets the average survival time value of the left half is significantly higher than the average value of the right half. The last column of this table indicates the computational times of out RL algorithm.

## Discussion

In this paper we have tackled the biological temporal ordering problem, defined as the problem of building a temporal ordering of a set of input samples, characterized by multi-dimensional data, so as to reflect the evolution and dynamics of a certain biological process. We have proposed a reinforcement learning based technique to address this problem, which is generally applicable to data sets containing instances represented by multi-dimensional data, that can be temporally ordered.

To experimentally evaluate our approach, we selected a series of data sets that have already been used in the literature [20], [21]. The results that we obtained are comparable, or in some cases even better than the results that were reported, so far. Firstly, we tested our technique on several time series gene expression data sets, in which the exact time of extraction of each sample is provided and the temporal orderings are known. The time series data sets we used belong to yeast and human cells, which are affected by various external conditions [38]–[40]. The first two data sets were also experimented on by Gupta and Bar-Joseph [20], while the third one was used in the work of Magwene *et al.* [21]. In order to quantify and compare our results with the ones existing in the cited studies, we have also introduced an evaluation measure, which expresses the degree in which samples are misplaced in a given ordering.

We note that our RL based algorithm obtained the correct orderings for *seven* out of the ten data sets. For three of the data sets (“yeast cells: heat shock”, “human cells - Wild type 2” and “human cells - Mutant 2”) our approach recovered the correct ordering, contrary to the one reported by the TSP approach introduced by Gupta and Bar-Joseph [20]. Moreover, for the situations when the RL recovered ordering is not the correct one, we remark that in the case of the “human cells - Mutant 1” experiment, our RL algorithm reported a slightly better solution than the TSP algorithm, in terms of the evaluation measure we defined. The only case in which our solution is less performant is the *Saccharomyces cerevisiae* yeast data set [39].

Secondly, we evaluated our RL based method on two cancer gene expression data sets: one composed of 28 glioblastomas and the second cotaining 22 anaplastic oligodendrogliomas [41]. Based on an intuitive correlation between the advancement of the disease and overall survival time of the patients (as cancer progresses, the life expectancy decreases), we have chosen the survival time as a measure of validation for the obtained temporal orderings. Although for the anaplastic oligodendrogliomas data set this correlation is stronger, we may state that in both cases, the retrieved orderings are, up to a certain extent, well correlated with the survival time.

Regarding the 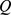-learning based approach that we introduced for solving the temporal ordering problem, we remark the following. Considering that the TO agent performs an intelligent action selection mechanism (the *look-ahead* procedure), the training process during an episode has a time complexity of 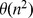, where 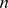 is the number of samples considered in the ordering process. Consequently, assuming that the number of training episodes is 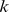, the overall complexity of the algorithm for training the TO agent is 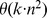. We mention that if the number 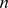 of the samples considered in the temporal ordering problem is large and consequently the state space becomes very large, in order to store the 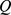 values estimates, we should use a function approximation method (e.g. neural network, support vector machine).

Concerning the computational time, we remark that our 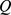-learning based approach has a complexity of 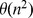, for each episode, while the TSP approximation algorithm used by Gupta and Bar-Joseph [20] finds the optimal ordering in 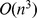 time, where 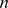 is the number of samples. As in the work of Magwene *et al.* [21] an asymptotic analysis of the time complexity is not given, we cannot provide a detailed comparison.

The methodology that we introduced is general, it can be used with various types of multi-dimensional biological data, not being restricted to microarray data. In this work, for the data pre-processing step, we use certain additional biological information (given correct ordering - for time series or survival time - for the cancer sets), but in case these are not available, other types of biological information may be employed, or, in certain situations, this step could even be neglected.

The solution to the temporal ordering problem can be used for a dual purpose. On the one hand, when given a set of data associated to a certain biological system, a temporal ordering of this data could offer new insights into the development of the considered processes. On the other hand, our approach could also be used to find the correct time points of some given new samples in a pre-ordered data set. One application for this is within the field of cancer research: assuming an ordered set of cancer patients, together with their overall survival time predictions are given, when new patients, with yet unknown survival times are added to the data set, a temporal ordering of the new set (including the new patients) could reveal important information regarding their life expectancies.

As disadvantages of our approach, we mention that the only way to reduce the noise that could affect the input data is the pre-processing step, that uses “a-priori” knowledge on the problem (i.e. the correct temporal ordering or the given overall survival time for the biological samples). Another drawback may be the fact that a large number of training episodes has to be considered for large problems (large values of 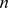) in order to obtain accurate results and this leads to a slow convergence. But, as the experimental results have shown, in order to speed up the convergence process, good local search mechanisms may be successfully used. Still, we think that the direction of using reinforcement learning techniques in solving the temporal ordering problem is worth being studied and further improvements can lead to more valuable results.

## Conclusions

In this paper we have approached, from a computational point of view, the biological temporal ordering problem and we proposed a reinforcement learning based approach for solving it. To our knowledge, this problem has not been addressed in the literature using reinforcement learning, so far. We have emphasized the potential of our proposal, highlighting its advantages and drawbacks.

We plan to extend the evaluation of the proposed RL model for some other data sets, to further test its performance. The efficiency of using different similarity measures between the samples, to consider different types of biological information, as well as the problem of noisy data will be analyzed in the future.

From a computational point of view, we will investigate possible improvements of the RL-based model by: using different reinforcement functions; adding various local search mechanisms in order to increase the model's performance; using function approximation methods (e.g. neural networks, support vector machines) to approximate the 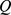 values, for the cases when the state space becomes very large; considering a decreasing 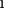-Greedy strategy for the action selection mechanism. An extension of the TO model to a distributed RL approach will be also considered.
