# Correction: Temporal Ordering of Cancer Microarray Data through a Reinforcement Learning Based Approach

**DOI:** 10.1371/annotation/21ae47e8-3ca1-41be-9262-4bc17eb445df

**Published:** 2013-10-09

**Authors:** Gabriela Czibula, Iuliana M. Bocicor, Istvan-Gergely Czibula

Several errors were introduced in the preparation of this article for publication.

In the last paragraph of "The proposed RL task for the TO problem" section of the "Our Approach. Methodology" portion of the article, there is an error in the fourth line. The word "Thorne" should be "π." This symbol displays correctly in the PDF.

In the "Synthetic Data" portion of the "Results" section, under the heading "RL model and results," there are two errors in the fourth line of the third paragraph:

-"1097/OLQ.1090b1013e318063c318734" should be "13000."

-The symbol "ô" should be " ε."

The correct phrase reads: "the number of training episodes is 13000; the modified ε-Greedy action selection mechanism was used with ε= 0.8." This phrase displays correctly in the PDF.

In the third paragraph of the "Conclusions" section, in the 5th line, "1-Greedy" should be "ε-Greedy." This error is also present in the PDF. 

